# Intracellular Ca^2+^ homeostasis and JAK1/STAT3 pathway are involved in the protective effect of propofol on BV2 microglia against hypoxia-induced inflammation and apoptosis

**DOI:** 10.1371/journal.pone.0178098

**Published:** 2017-05-23

**Authors:** Yan Lu, Yuechao Gu, Xiaowei Ding, Jiaqiang Wang, Jiawei Chen, Changhong Miao

**Affiliations:** 1 Department of Anesthesiology, Fudan University Shanghai Cancer Centre, Fudan University, Shanghai Shi, P.R. China; 2 Department of Oncology, Shanghai Medical College, Fudan University, Shanghai Shi, P.R. China; Uniformed Services University, UNITED STATES

## Abstract

**Background:**

Perioperative hypoxia may induce microglial inflammation and apoptosis, resulting in brain injury. The neuroprotective effect of propofol against hypoxia has been reported, but the underlying mechanisms are far from clear. In this study, we explored whether and how propofol could attenuate microglia BV2 cells from CoCl_2_-induced hypoxic injury.

**Methods:**

Mouse microglia BV2 cells were pretreated with propofol, and then stimulated with CoCl_2_. TNF-α level in the culture medium was measured by ELISA kit. Cell apoptosis and intracellular calcium concentration were measured by flow cytometry analysis. The effect of propofol on CoCl_2_-modulated expression of Ca^2+^/Calmodulin (CaM)-dependent protein kinase II (CAMKIIα), phosphorylated CAMKIIα (pCAMKIIα), STAT3, pSTAT3^Y705^, pSTAT3^S727^, ERK1/2, pERK1/2, pNFκB(p65), pro-caspase3, cleaved caspase 3, JAK1, pJAK1, JAK2, pJAK2 were detected by Western blot.

**Results:**

In BV2 cell, CoCl_2_ treatment time-dependently increased TNF-α release and induced apoptosis, which were alleviated by propofol. CoCl_2_ (500μmol/L, 8h) treatment increased intracellular Ca^2+^ level, and caused the phosphorylation of CAMKIIα, ERK1/2 and NFκB (p65), as well as the activation of caspase 3. More importantly, these effects could be modulated by 25μmol/L propofol via maintaining intracellular Ca^2+^ homeostasis and via up-regulating the phosphorylation of JAK1 and STAT3 at Tyr705.

**Conclusion:**

Propofol could protect BV2 microglia from hypoxia-induced inflammation and apoptosis. The potential mechanisms may involve the maintaining of intracellular Ca^2+^ homeostasis and the activation of JAK1/STAT3 pathway.

## Introduction

Perioperative acute ischemic/hypoxic stroke is one of the most serious complications during many surgical procedures, and it is associated with high morbidity and mortality. In the central nervous system, microglia, acting as the unique resident immune cells, could be activated following brain ischemia/hypoxia[1, 2]. Its activation can lead to the production and release of multiple pro-inflammatory cytokines, such as interleukin (IL)-1 and tumor necrosis factor-alpha (TNF-α). TNF-α, the major pro-inflammatory cytokine, may serve as a marker for inflammation. In the central nervous system, TNF-α-induced inflammation may damage neural cells and the blood-brain barrier[3]. In addition, ischemia/hypoxia may also cause microglial apoptosis, leading to impaired immune response[4].

Intracellular calcium, one of the most important second messenger, played a pivotal role in multiple physical procedures and noxious stimulation can induce an increase of Intracellular Ca^2+^. Previous studies reported that hypoxia could induce intracellular Ca^2+^ overload[5, 6]. Microglial cells are the resident immune cells in the central nervous system and many of their physiological functions are linked to Ca^2+^ signaling[7]. Previous studies showed that morphological changes, migration, proliferation, and secretion of cytokines and reactive oxygen species were related to intracellular Ca^2+^ overload [8, 9]. As the major isoform of Ca^2+^ /calmodulin-dependent protein kinase (CaMK) in brain, CaMKIIα is highly sensitive to intracellular Ca^2+^ levels. Recent data suggested that the activation of CaMKIIα is directly associated with the production of pro-inflammatory cytokines, such as TNF-α and IL-1β and the mechanism may involve phosphorylating and upregulating the expression of several down-stream proteins[10, 11]. Previous studies have showed that ERK 1/2, nuclear factor kappa B (NF-κB) are involved in Ca^2+^-mediated TNF-α release[12].

Janus kinase-signal transducers and activators of transcription (JAK-STAT) signal pathway has been reported to be involved in the immune response of numerous cytokines[13]. Activation of STAT3 and JAK has been shown to mediate ischemic and several pharmacological postconditioning[14–16]. Phosphorylation of JAKs may regulate STAT3’s activity by phosphorylating STAT3 on tyrosine705 and serine727 residues. Reduced phosphorylation of STAT3 at Ser727 is usually correlated with increased phosphorylation at Tyr705[17, 18]. A previous study showed that JAK1 and STAT3 are activated in neurons, astrocytes and microglia after focal cerebral infarction, and may provide neuroprotection in the acute phase of ischemia[14].

Propofol (2,6-diisopro-pylphenol) is a widely used intravenous anesthetic. In addition to sedative property, propofol has been proved to exert beneficial effects in multiple organs and tissues, such as cardiovascular system[19], respiratory system[20] and urinary system[21]. In brain injury model, a previous study showed that propofol could protect central nervous system from hypoxic injury via improving the oxygen supply and maintaining oxygen metabolism[22]. Another study also showed that propofol protects the brain through maintaining intracellular Ca^2+^ homeostasis[23]. However, other groups reported propofol-induced neuroinflammation and cell death in young rodents[24]. An animal study showed that propofol protected against focal cerebral ischemia via inhibition of microglia-mediated proinflammatory cytokines[25]. However, the mechanism of propofol inhibiting hypoxia-induced microglial activation is yet to be investigated. In this study, we used cobalt chloride (CoCl_2_) to build an in vitro hypoxic model and aimed to clarify whether and how propofol attenuates CoCl_2_-induced BV2 cell injury.

## Materials and methods

### Cell culture and reagents

BV2 microglia cells were obtained from GuangZhou Jennio Bio- tech. The cells were cultured in DMEM (Sigma–Aldrich, Shanghai, China) with 10% fetal bovine serum (Gibco, Life technologies, USA), 100 units/ml penicillin (sigma) and 100 μg/ml streptomycin (sigma) in an incubator containing 5% CO_2_ at 37°C. BV2 cells were sub-cultured when reaching 90% confluence. The eighth passage was used in the present study.

Propofol (Sigma, St. Louis, MO, USA), calcium chelator BAPTA-AM (Sigma, St. Louis, MO, USA), CAMKIIα inhibitor KN93 (Sigma, St. Louis, MO, USA), ERK inhibitor U0126 and JAK1 inhibitor INCB039110 were dissolved in DMSO (Sigma, St. Louis, MO, USA). The final concentration of DMSO was adjusted to 0.01% for each solution to avoid possible nonspecific effects. A 500mM stock solution of cobalt chloride (CoCl_2_) was prepared by dissolving CoCl_2_ powder (Sigma-Aldrich, Shanghai, China) in serum-free DMEM.

### Experiment design

To determine the appropriate treatment condition, BV2 cells were treated with 500 μM CoCl_2_ (sigma) for 0, 1, 2, 4, 8 and 12 h respectively. Cells culture in DMEM, without any treatment, served as the control group. The time with maximal effects on TNF-α production was used as the appropriate treatment condition. Then cells were pre-treated with different concentrations of propofol (sigma) (5 μmol/L, 25 μmol/L, 50 μmol/L and 100 μmol/L), followed by CoCl_2_ (500 μM, 8 h) treatment, and the concentration with maximal protective effects was determined. In the following experiments, the optimal concentration of CoCl_2_ and propofol were used to investigate the potential mechanisms.

### Enzyme-linked immunosorbent assay (ELISA)

To determine TNF-α concentration in the culture medium, a TNF-α sandwich ELISA kit from BioSource International Inc was used according to the manufacturer’s instructions. The absorbance at 450 nm was measured with a microplate reader. The range of detection was from 0 to 1000 pg/ml.

### Measurement of intracellular free Ca^2+^ concentration

Intracellular free Ca^2+^ concentration was detected by the fluorescent dye Fluo-3 AM (Beyotime biotechnology, shanghai, China). The Fluo-3 could specifically bind to the Ca^2+^ and has a strong fluorescence with an excitation wavelength of 488 nm. After designed treatment, BV2 cells were harvested and washed twice with PBS, then resuspended with fluo-3 AM (5 μM) for 30 min in the dark. Intracellular Ca^2+^ was detected by Flow cytometer at 488 nm excitation wavelength.

### Cell apoptosis analysis

Cell apoptosis was detected by propidium iodide (PI) and annexin V staining according to the manufacture’s instructions. Briefly, after designed treatment, cells were harvested and stained with annexin V and PI. Then stained cells were analyzed by flow cytometry (Cytomics FC 500 MPL, Beckman Coulter).

### Western blot analysis

After designed treatment, whole cell extracts were collected and lysed with lysis buffer. Equivalent amounts of protein in each sample (about 40 μg) were separated by 10% or 12% SDS-PAGE, and transferred to polyvinylidene difluoride (PVDF) membranes. The membranes were blocked with 5% skim milk for 1 h, and then incubated with appropriate primary antibody at 4°C overnight. After that, the membranes were washed and incubated with secondary antibodies conjugated with horseradish peroxidase (HRP) for 1 h at room temperature. The protein bands were developed with the chemiluminescent reagents (Millipore, MA, USA). Antibodies to Stat3 (4904, 1:1000), ERK1/2 (9102, 1:1000), pERK1/2 (4370, 1:500), pNFκB(p65) (3033, 1:500), cleaved caspase 3 (9665, 1:1000), pro-caspase 3 (9665, 1:1000), JAK1 (3332, 1:1000), pJAK1 (74129, 1:500), JAK2 (3771, 1:1000) were obtained from cell signaling technology. Antibodies to pStat3^Y705^ (CY6566, 1:500), pStat3^S727^ (CY6500, 1:500), pJAK2 (CY6570, 1:500) were obtained from Abways technology. Antibodies to CAMKIIα (ab131468, 1:1000), pCAMKIIα (ab5683, 1:500) were obtained from Abcam. The antibody to β-actin (6008-1-lg, 1:1000) was obtained from proteintech. β-actin served as loading control and the intensity of each protein band was normalized with that of β-actin.

### Statistical analysis

Results were expressed as mean ± SD, and data were obtained from at least 5 separately performed experiments. Differences between groups was determined by one-way ANOVA followed by the Newman–Keuls test using the InStat statistical program (GraphPad Software, San Diego, CA, USA). All results were considered statistically significant at a value of p< 0.05.

## Results

### Effects of propofol on CoCl_2_ induced TNF-α generation

**Fig 1:**
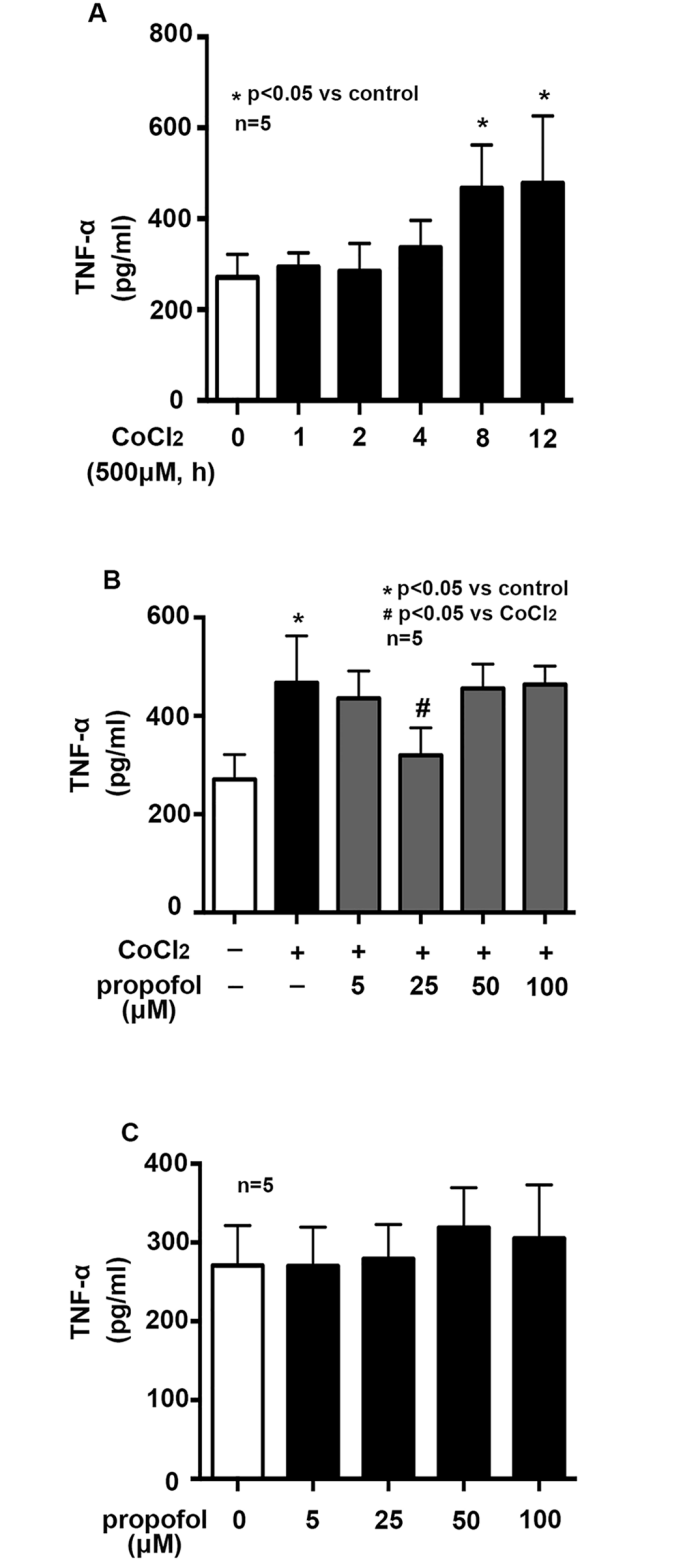
Propofol attenuates CoCl_2_-induced TNF-α generation. A, In BV2 cells, 500 μM CoCl_2_ treatment induced TNF-α generation in a time-dependent manner, and 8h treatment significantly increased TNF-α generation. B, propofol attenuated CoCl_2_-induced TNF-α generation in a suitable concentration. 25μM propofol significantly reduced TNF-α generation. C, propofol alone had no effect on TNF-α generation. (* p < 0.05 vs. control, # p < 0.05 vs. CoCl_2_ treatement, n = 5, Data were shown as mean ± SD).

In BV2 cells, 500μM CoCl_2_ treatment induced TNF-α production in a time-dependent manner. Compared with control, we found that treatment of cells with 500μM CoCl_2_ for 8 hours induced a notably generation of TNF-α (Fig 1A, p< 0.05). To observe the effect of propofol on CoCl_2_ induced TNF-α generation, BV2 cells were pretreated with propofol for 2 h with different concentration (5, 25, 50, 100μM) after CoCl_2_ treatment. As shown in Fig 1B, 25μM propofol significantly reduced the CoCl_2_-induced generation of TNF-α. We found that propofol alone had no effect on TNF-α generation (Fig 1C). Thereafter, 8 h treatment of 500μM CoCl_2_ and 25μM of propofol pretreatment for 2h were used in the following experiments to study the signaling pathway involved in the protective effects of propofol.

### Effects of propofol on CoCl_2_ induced cell apoptosis

**Fig 2:**
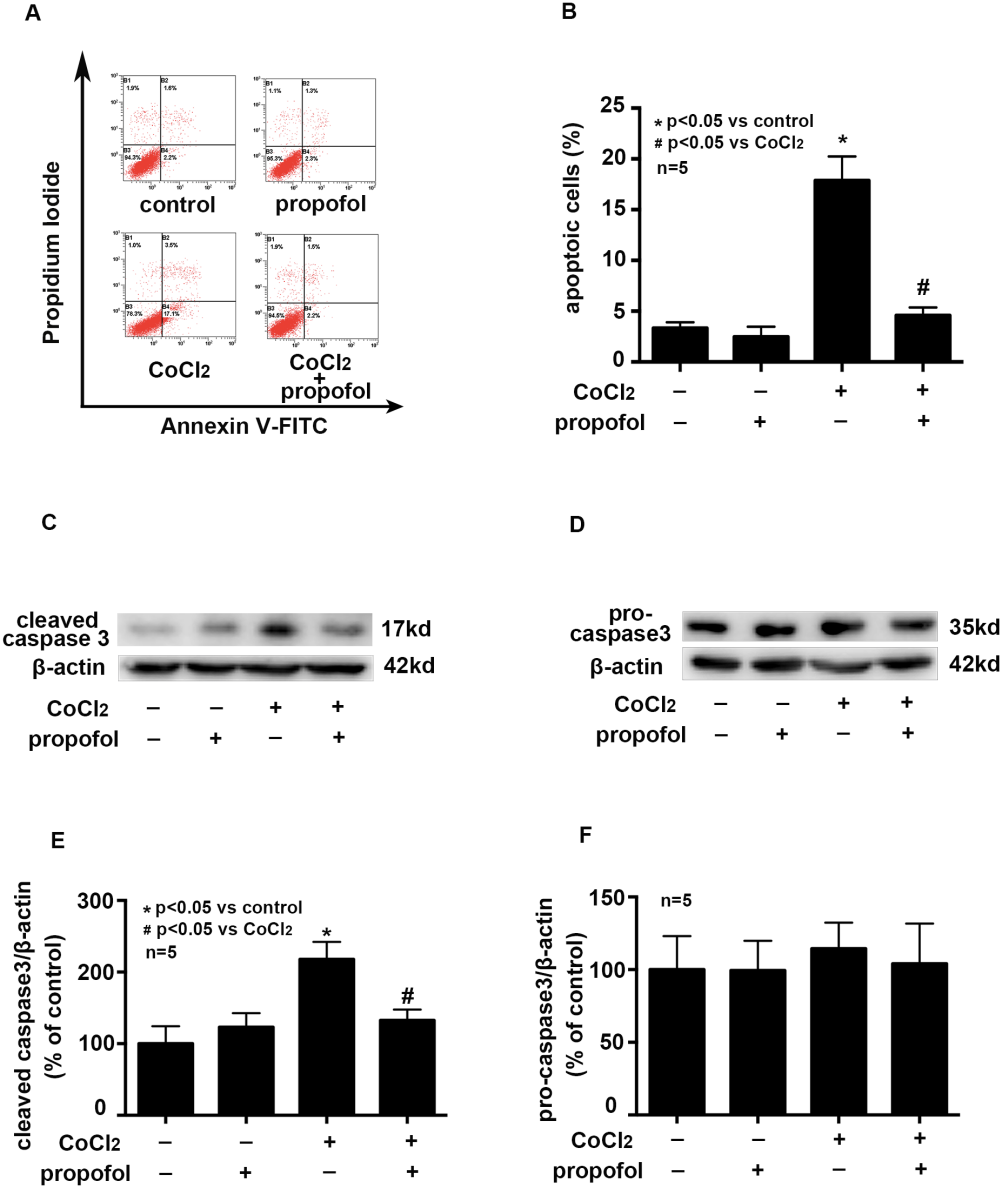
Propofol attenuated CoCl_2_-induced cell apoptosis. A and B, CoCl_2_-increased cell apoptosis was attenuated by propofol. C and E, CoCl_2_-induced cleaved caspase 3 overexpression was attenuated by propofol. D and F, CoCl_2_ and propofol treatment had no effect on the expression of pro-caspase 3. (* p < 0.05 vs. control, # p < 0.05 vs. CoCl_2_ treatement, n = 5, Data were shown as mean ± SD).

Compared with control, CoCl_2_ (500μM, 8 h) treatment increased the percentage of apoptotic cells from 3.33 ± 0.33% to 17.87 ± 1.37% and increased the expression of cleaved caspase 3 by 2.17 ±  0.13 folds (Fig 2, p < 0.05). More importantly, we found 25μM propofol reduced the percentage of apoptotic cells from 17.87 ± 1.37% to 4.56 ± 0.47% and decreased the expression of cleaved caspase 3 by 60.9 ± 0.09% (Fig 2, p < 0.05). However, CoCl_2_ and propofol treatment had no effect on the expression of pro-caspase 3 (Fig 2). Our data suggested that CoCl_2_ induced apoptosis, which could be inhibited by propofol. Besides, 25μM propofol treatment alone had no effect on cell apoptosis.

### Effects of propofol on CoCl_2_ increased intracellular Ca^2+^ level and the phosphorylation of CAMKIIα, ERK, NF-κB

**Fig 3:**
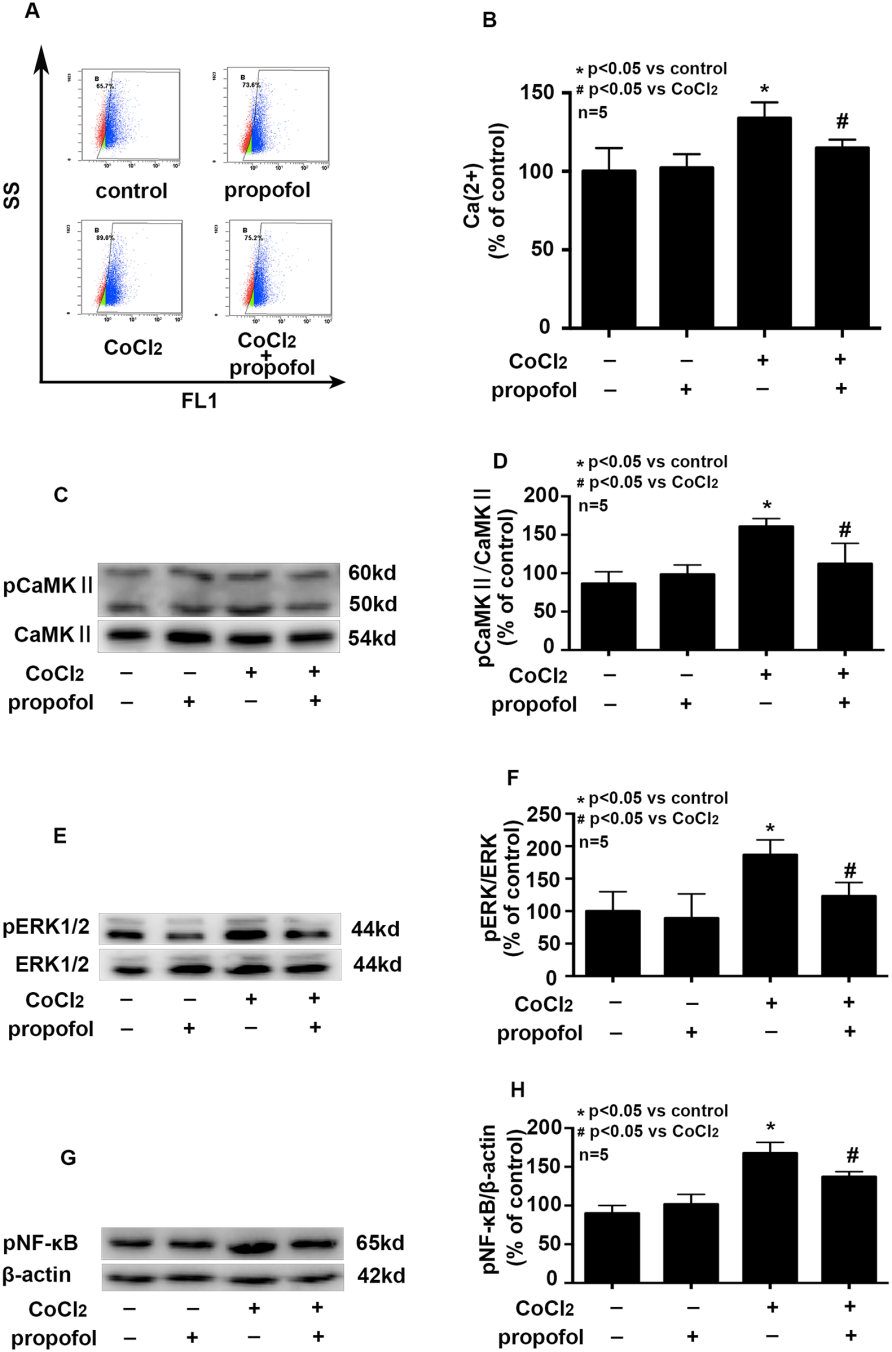
Effects of propofol on CoCl_2_ induced the destruction of intracellular Ca^2+^ homeostasis and the phosphorylation of CAMKIIα, ERK, NF-κB. A and B, CoCl_2_-increased intracellular Ca^2+^ concentration was attenuated by propofol. C, D, E, F, G and H, CoCl_2_-induced the phosphorylation of CAMKIIα, ERK and NF-κB were attenuated by propofol. (* p < 0.05 vs. control, # p < 0.05 vs. CoCl_2_ treatement, n = 5, Data were shown as mean ± SD).

We found that compared with control, CoCl_2_ treatment increased cytoplasmic Ca^2+^ concentration by 1.33 ± 0.06 folds (Fig 3A and 3B, P < 0.05), and propofol pretreatment attenuated CoCl_2-_induced intracellular Ca^2+^ overload (Fig 3A and 3B, p <0.05).

We demonstrated that, after CoCl_2_ treatment, the phosphorylation of CAMKIIα, ERK, NF-κB proteins enhanced compared with control (Fig 3C, 3D, 3E, 3F, 3G and 3H, p<0.05). More importantly, we found propofol pretreatment down-regulated the phosphorylation of CAMKIIα (Fig 3C and 3D, p<0.05), ERK (Fig 3E and 3F, p<0.05) and NF-κB (Fig 3G and 3I, p<0.05). Besides, 25μM propofol treatment alone had no such effects.

### Effects of propofol on CoCl_2_ modulated JAK/STAT3 pathway

**Fig 4:**
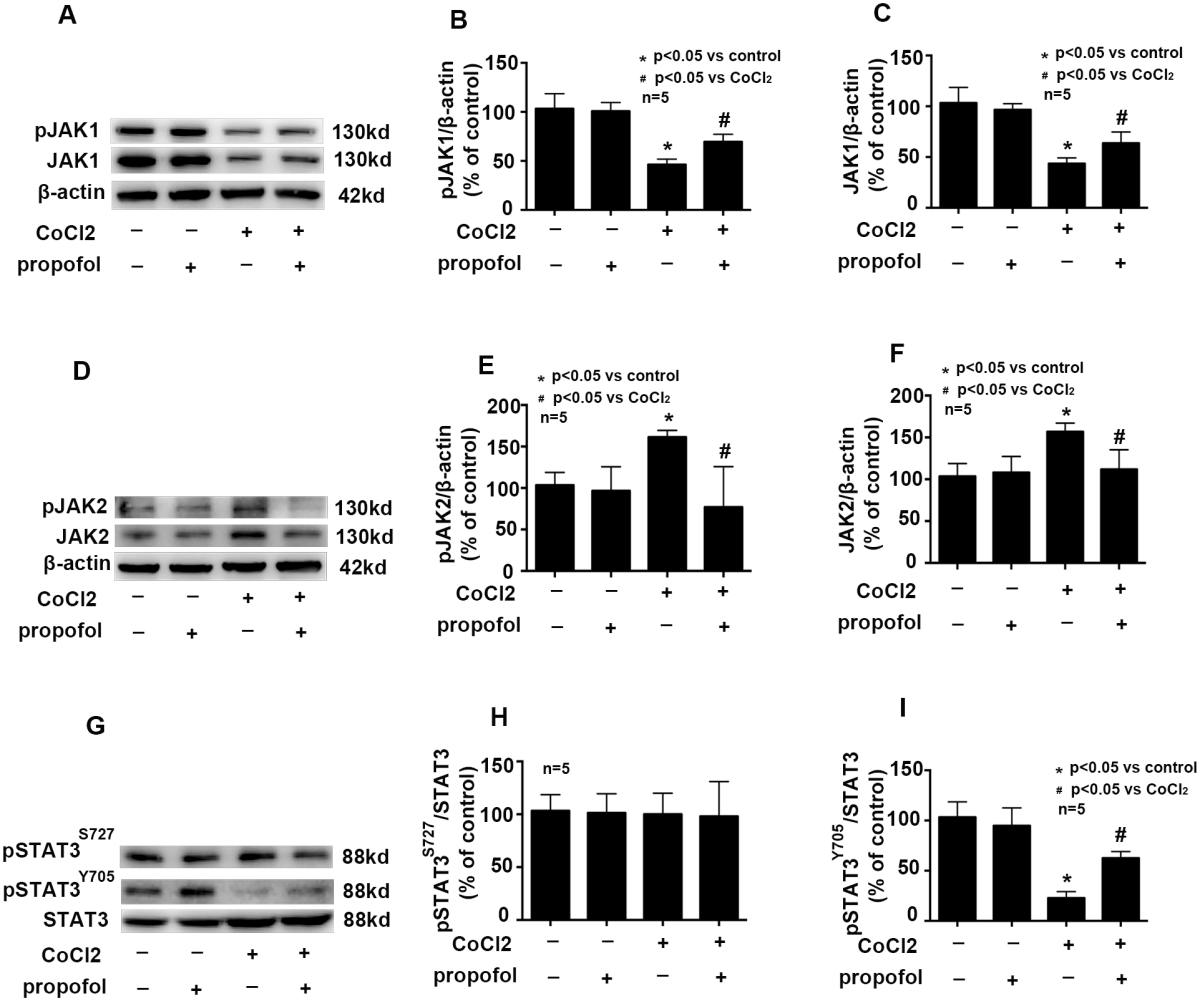
Effects of propofol on CoCl_2_ modulated JAK/STAT3 pathway. A, B and C, CoCl_2_-decreased the expression and phosphorylation of JAK1 were attenuated by propofol. D, E, and F, CoCl_2_-increased the expression and phosphorylation of JAK2 were attenuated by propofol. G, H and I, CoCl_2_-decreased the phosphorylation of STAT3 at Tyr705 were attenuated by propofol. CoCl_2_ and propofol had no effect on the phosphorylation of STAT3 at Ser727. (* p < 0.05 vs. control, # p < 0.05 vs. CoCl_2_ treatement, n = 5, Data were shown as mean ± SD).

Previous studies have shown that after focal cerebral infarction, the activation of JAK1 and STAT3 in microglia may provide neuroprotection in the acute phase of ischemia[14]. Here, we found that CoCl_2_ treatment decreased the expression and phosphorylation of JAK1 (Fig 4A, 4B and 4C, p<0.05), but increased the expression and phosphorylation of JAK2 (Fig 4D, 4E and 4F, p<0.05). Besides, CoCl_2_ treatment decreased the phosphorylation of STAT3 at Tyr-705, but had no effect on the phosphorylation of STAT3 at Ser-727 (Fig 4G, 4H and 4I, p<0.05). More importantly, we found propofol attenuated these CoCl_2_-modulated effects (Fig 4, p<0.05).

### Effects of propofol, BAPTA-AM, KN93, U0126, INCB039110 on CoCl_2_ induced TNF-α generation

**Fig 5:**
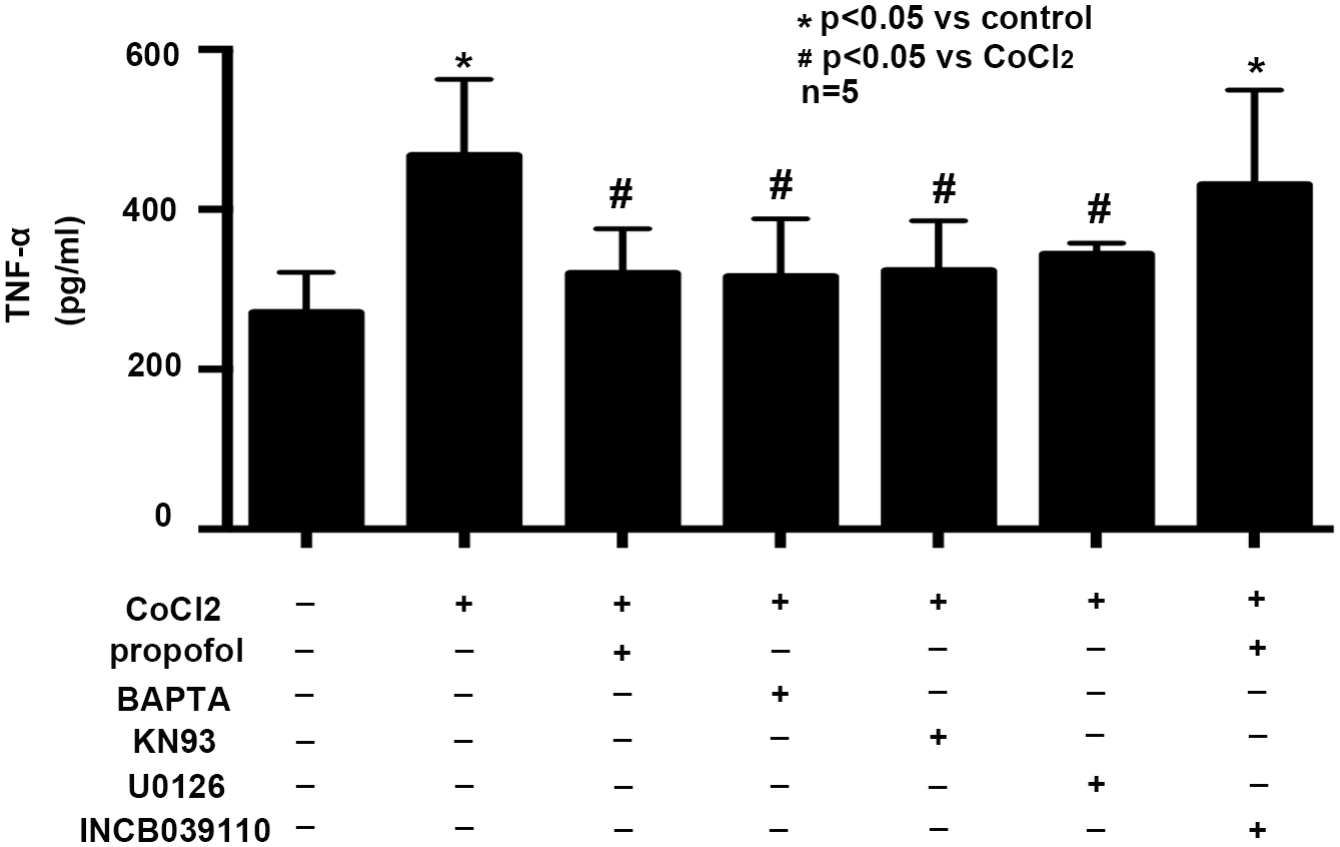
Effects of propofol, BAPTA-AM, KN93, U0126, INCB039110 on CoCl_2_ induced TNF-α generation. CoCl_2_ treatment induced TNF-α generation was attenuated by propofol and BAPTA-AM, KN93, U0126. The effect of propofol on TNF-α generation was reversed by INCB039110. (* p < 0.05 vs. control, # p < 0.05 vs. CoCl_2_ treatement, n = 5, Data were shown as mean ± SD).

To confirm the role of Ca^2+^ homeostasis and the phosphorylation of CAMKIIα, ERK and NF-κB on the production of TNF-α, BV2 cells were pretreated with calcium chelator BAPTA-AM, CAMKIIα inhibitor KN93, or ERK inhibitor U0126 followed by CoCl_2_ treatment. We found that compared with CoCl_2_ treatment, BAPTA-AM, KN93 and U0126 significantly decreased the generation of TNF-α, which was similar to propofol treatment. Further, to confirm the role of JAK1/STAT3 pathway in the anti-inflammatory property of propofol, BV2 cells were pretreated with propofol and selective JAK1 inhibitor INCB039110 followed by CoCl_2_ treatment. We found INCB039110 alleviated the effect of propofol, resulting in increased production of TNF-α (Fig 5, p<0.05).

### Effects of propofol, BAPTA-AM, KN93, U0126 on CoCl_2_ induced the phosphorylation of CAMKIIα, ERK, NF-κB

**Fig 6:**
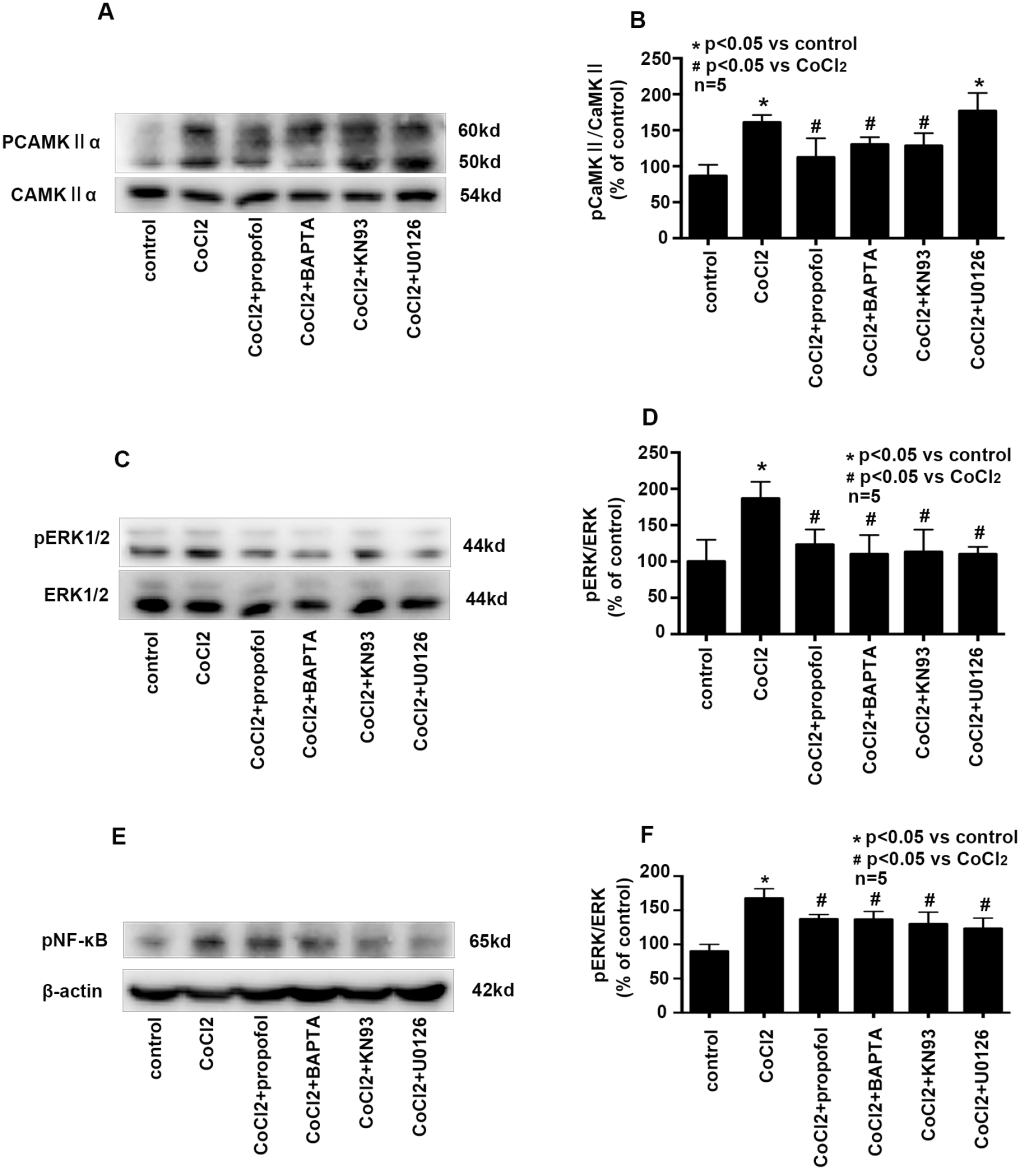
Effects of propofol, BAPTA-AM, KN93, U0126 on CoCl_2_ induced the phosphorylation of CAMKIIα, ERK, NF-κB. CoCl_2_-induced the phosphorylation of CAMKIIα, ERK and NF-κB were attenuated by propofol, BAPTA-AM, KN93 and U0126. (* p < 0.05 vs. control, # p < 0.05 vs. CoCl_2_ treatement, n = 5, Data were shown as mean ± SD).

To confirm the role of the phosphorylation of CAMKIIα, ERK and NF-κB in the protective effects of propofol against CoCl_2_ treatment, BV2 cells were pretreated with calcium chelator BAPTA-AM, CAMKIIα inhibitor KN93, or ERK inhibitor U0126 followed by CoCl_2_ treatment. We found that compared with CoCl_2_ treatment, BAPTA-AM and KN93 significantly decreased the phosphorylation of CAMKIIα, which was similar with propofol treatment. However, U0126 pretreatment had no effect on the phosphorylation of CAMKIIα (Fig 6A and 6B, p<0.05). Compared to CoCl_2_ treatment, BAPTA-AM, KN93 and U0126 significantly decreased the phosphorylation of ERK (Fig 6C and 6D, p<0.05) and NF-κB (Fig 6E and 6F, p<0.05), which was similar to propofol treatment.

### Effects of propofol, BAPTA-AM, KN93 and U0126 on CoCl_2_ induced cell apoptosis

**Fig 7:**
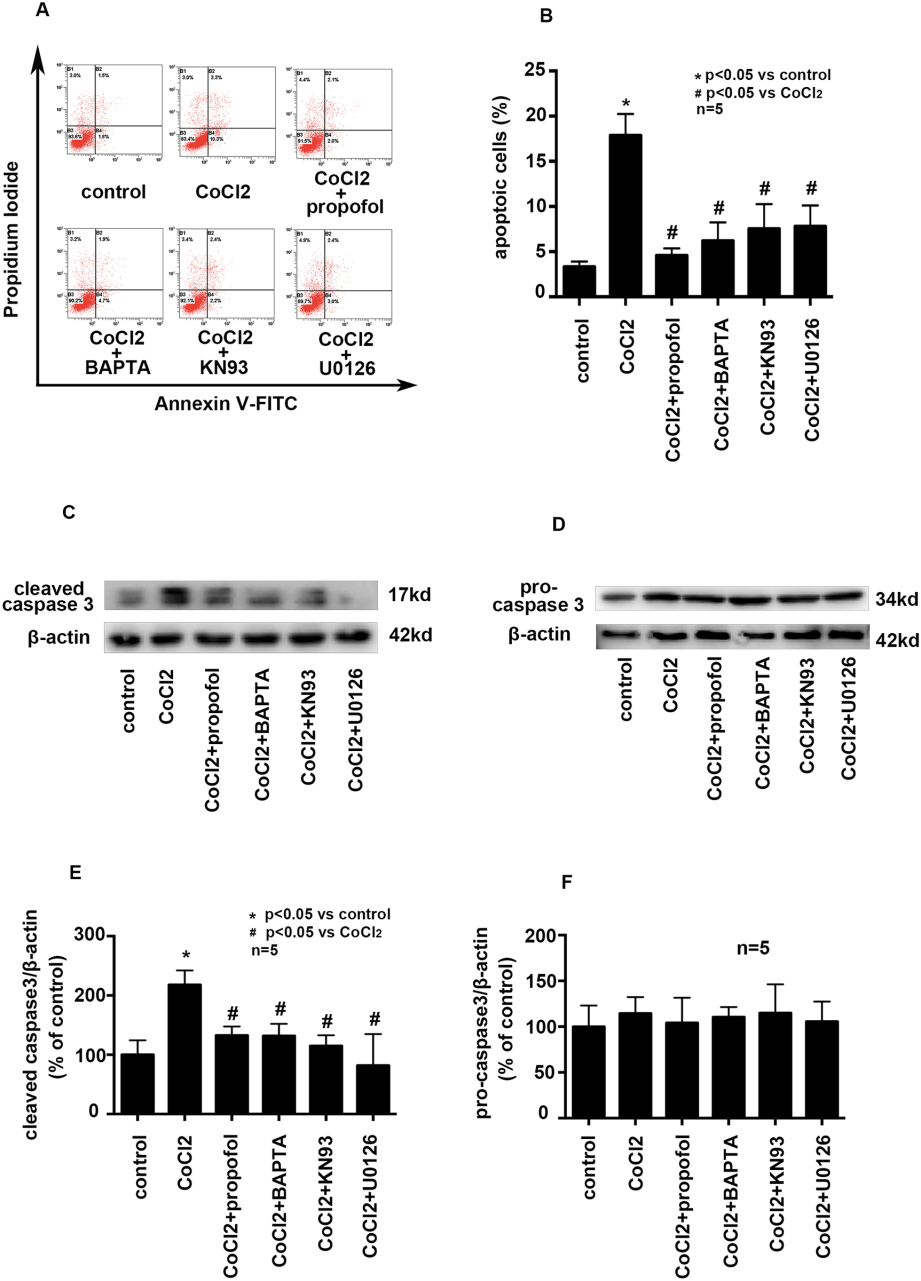
Effects of propofol, BAPTA-AM, KN93, U0126 on CoCl_2_ induced cell apoptosis. A and B, CoCl_2_-increased cell apoptosis was attenuated by propofol, BAPTA-AM, KN93 and U0126. C and E, CoCl_2_-induced cleaved caspase 3 overexpression was attenuated by propofol, BAPTA-AM, KN93 and U0126. D and F, CoCl_2_, propofol, BAPTA-AM, KN93 and U0126 had no effect on the expression of pro-caspase 3. (* p < 0.05 vs. control, # p < 0.05 vs. CoCl_2_ treatement, n = 5, Data were shown as mean ± SD).

To confirm the role of Ca^2+^ homeostasis and the phosphorylation of CAMKIIα, ERK and NF-κB pathway on cell apoptosis, BV2 cells were pretreated with calcium chelator BAPTA-AM, CAMKIIα inhibitor KN93, or ERK inhibitor U0126 followed by CoCl_2_ treatment. Compared with CoCl_2_ treatment, BAPTA could reduce the percentage of apoptotic cells from 17.87 ± 1.37% to 6.2 ± 1.17%, KN93 could reduce the percentage of apoptotic cells from 17.87 ± 1.37% to 7.53 ± 1.57% and U0126 could reduce the percentage of apoptotic cells from 17.87 ± 1.37% to 7.8 ± 1.33% (Fig 7A and 7B, p<0.05). We also found that, compared with CoCl_2_ treatment, BAPTA decreased the expression of cleaved caspase 3 by 85.9 ± 0.02%, KN93 decreased the expression of cleaved caspase 3 by 103 ± 0.17%, and U0126 decrease the expression of cleaved caspase 3 by 136 ± 0.34% (Fig 7C and 7E, P<0.05). However, Propofol, BAPTA-AM, KN93 and U0126 pretreatment had no effect on the expression of pro-caspase 3 (Fig 7D and 7F, p<0.05).

## Discussion

In the present study, we found that CoCl_2_ treatment could disrupt the intracellular Ca^2+^ hemostasis, and phosphorylate CAMKIIα, ERK and NF-κB, resulting in the production of TNF-α and apoptosis. More importantly, we found propofol could protect BV2 cells from CoCl_2_-induced injury via maintaining the intracellular Ca^2+^ hemostasis and via inhibiting Ca^2+^-related signaling pathway, as well as via up-regulating JAK1/STAT3 pathway.

Emerging evidences have suggested that transient cerebral ischemia/hypoxia may lead to microglial malfunction and microglial activation may lead to neurodegeneration via inflammation or cell injury[26–28]. Here, we used CoCl_2_-treated BV2 cell line to mimic the cerebral hypoxia in vivo. We found CoCl_2_ treatment induced the release of TNF-α (Fig 1). TNF-α is a main pro-inflammatory cytokine, and is mainly produced by activated microglia during the neuroinflammation. Growing evidence showed that the overproduction of TNF-α by microglia contributes to pathophysiological changes observed in various neurologic diseases and brain injury [3, 29]. In addition to inflammation caused by ischemia/hypoxia, we also found CoCl_2_ treatment induced cell apoptosis (Fig 2).

As mentioned above, intracellular calcium plays a key role in maintain cell function and noxious stimulations can induce an increase of intracellular Ca^2+^ concentration. Besides, intracellular Ca^2+^ overload could upregulate the expression of several down-stream proteins. In the situation of ischemic myocardial stress response, CAMKIIα could activate ERK and NF-κB, thus regulating inflammation and injury[12]. In our study, as shown in Fig 3, CoCl_2_ treatment disrupted the intracellular Ca^2+^ hemostasis and inducing the phosphorylation of CAMKIIα, ERK and NF-κB.

JAK/STAT signaling plays an essential role in promoting and modulating immune and inflammatory processes[30]. Activation of the JAK/STAT pathway has been associated with pathological conditions such as cerebral ischemia, traumatic brain injury and brain inflammation[31–33]. In the central nervous system, STAT1, STAT3 and STAT6 play an important role during brain development. It has been demonstrated that STAT3 plays critical roles in promoting the survival of neurons under pathological conditions[34]. It is known that STAT3 is activated by JAK1 and JAK2 in neuron cell lines[35]. As shown in Fig 4, we found CoCl_2_ affected the phosphorylation of both JAK1 and JAK2. In addition, STAT3 function is controlled by phosphorylation at two sites: phosphorylation of Tyr705 by JAK leads to STAT3 transcriptional activation whereas the role of the second phosphorylation site (Ser727) varies depending on cell type and pathological conditions[36]. Besides, Ser727 phosphorylation was considered a negative regulatory mechanism of STAT3 activity[37]. A previous study showed that JAK1 and STAT3 are activated in neurons, astrocytes and microglia after focal cerebral infarction, and may provide neuroprotection in the acute phase of ischemia[14]. As shown in Fig 4, our study was in line with previous theory.

The neuroprotective effects of propofol have been extensively studied in previous investigations[38]. In this study, our data suggested that 25μM propofol inhibited microglial activation and suppresses cytokine release as well as apoptosis, which were induced by hypoxia injury. And our data is consistent with the previous study, which found that propofol suppressed hypoxia/reoxygenation-induced apoptosis in HBVSMC[39]. In patients, the effect site (brain) concentrations of propofol have been reported to range from about 2 to 6 μg/mL, which are equivalent to 11 to 33μM. In BV2 microglial cells, 30μM propofol was considered a clinically relevant concentration[40]. Therefore, in our study, the effective concentration of propofol was clinically relevant. Further, the underlying mechanism may involve maintaining intracellular Ca^2+^ hemostasis. Because we used BAPTA-AM, a calcium chelator, and found the effect of BAPTA-AM was similar to that of propofol. Consistently, animal study has showed that propofol exert neuroprotective effect via preventing calcium-induced mitochondrial swelling[41]. Besides, the JAK1/STAT3 pathway was also involved in the protective mechanism. In cervical cancer cells, previous study showed that propofol could enhance cisplatin-induced apoptosis via EGFR/JAK2/STAT3 pathway[42]. However, in our study, we found propofol could exert protective effects via activating JAK1, and inducing the phosphorylation of STAT3 at Tyr-705. Consistently, we used INCB039110, a selective JAK1 inhibitor, and found it could reverse the effect of propofol.

There are some limitations in this study. Firstly, the study was carried out in cultured cell line. It is noted that the data obtained from cell line may differ from those from primary culture and animal studies, and we are planning to perform the experiment in the mice model to validate our findings. Secondly, we found the beneficial effects of propofol were mediated via activating JAK1/STAT3 pathway, but we didn’t exam how JAK1/STAT3 pathway was activated. Future experiments are required to address this issue.

In summary, our study identified that propofol could exhibit anti-neuroinflammatory activity in vitro by suppressing the pro-inflammatory mediators from CoCl_2_-induced BV2 microglial cells and in turn prevents neuronal from further damage. Additionally, these beneficial effects were mediated via inhibiting cellular Ca^2+^ overload, modulating the phosphorylation of CaMKII, ERK and NF-κB, and activating JAK1/STAT3 pathway.
